# Magnetic resonance imaging, water relation, gene expression and biochemical data for analysis of the effects of water stress on potato plant functioning and tuber development and quality

**DOI:** 10.1016/j.dib.2024.110891

**Published:** 2024-09-03

**Authors:** Maja Musse, Sylvain Challois, Ghina Hajjar, Stéphane Quellec, Aël Radovcic, Nusrat Ali, Solenne Berardocco, Doriane Dumont, Patricia Laugier, Patrick Leconte, Aurélien Carrillo, Christophe Langrume, Lydia Bousset-Vaslin, Bastien Billiot, Franck Jamois, Carole Deleu, Laurent Leport

**Affiliations:** a UR OPAALE, INRAE, 17, avenue Cucillé, 35044 Rennes, France; b UMR IGEPP, INRAE, Institut Agro Rennes-Angers, Université de Rennes, La Motte au Vicomte, 35650 Le Rheu, France; c Centre Mondial de l'Innovation Roullier – Timac Agro International, 35400 Saint Malo, France; d MetaboHUB-Grand-Ouest, Rennes, France; e UR PSH, INRAE, Site Agroparc, Avignon 84914, France

**Keywords:** *Solanum tuberosum*, MRI, *In situ* study, Abiotic stress, Drought, Internal default, Rust spot

## Abstract

The data presented in this paper include the original and processed MRI images acquired with a 1.5 T whole-body MRI scanner, describing the growth kinetics, spatialization and appearance of internal defaults of individual tubers of potato plants (Rosanna cultivar of *Solanum tuberosum*) grown in pots in a semi-controlled environment and exposed to two water regimes. The 2 conditions were a well-watered regime, in which soil moisture was maintained at 70 % of field capacity, and a variable water deficit regime, in which soil moisture was reduced to 20 % of field capacity several times during tuber growth, followed each time by a few-day period of rehydration to 70 % of field capacity. These data are supplemented by physiological, biochemical and gene expression data obtained from the leaves and tubers of additional potato plants grown under the same conditions. All these data contribute to highlight the long-term effects of water stress on plant functioning with a particular focus on the growth kinetics, spatialization and quality of potato tubers. The dataset presented here is related to the research article entitled “Growth kinetics, spatialization and quality of potato tubers monitored *in situ* by MRI - long-term effects of water stress”. It is made publicly available to enable extended analyzes. It is a useful resource for biologists, agronomists and breeders interested in the potato crop, as well as for researchers interested in developing new imaging methods. All data can be used to improve databases on development and quality of tubers and to feed and validate mathematical models.

Specifications Table

**Table:** 

Subject	Agricultural and Plant Sciences
Specific subject area	*MRI, water relation, gene expression and biochemical dataset related to potato plant under optimal and water-stress conditions*
Type of data	image; Avizo project; table
Data collection	MRI images: 1.5 T scanner (Avanto, Siemens). Software: SYNGO (Siemens, version VB17) for image acquisition and Avizo 3D Pro (FEI Company, version 2021.2) for image processing. Electronic precision balance (0.1 mg) (leaf water deficit and tuber fresh weight), WP4C dewpoint potential meter (leaf water potential), Horiba LAQUAtwin-pH-22 m (tuber pH) and Ag/AgCl reference electrode radiometer analytical E21M002 and radiometer analytical platinum plate electrode, with a Voltcraft VC850 multimeter (tuber Eh). Microplate reader (starch analysis after enzymatic digestion); Agilent technology gas chromatography system (sugar analysis). Leaf gene expression: qRT-PCR using a Universal SYBR Green Supermix (Bio-Rad) in a real-time PCR detection system (Bio-Rad).
Data source location	Potato plants were grown under semi-controlled conditions in a greenhouse at UMR IGEPP, INRAE, Le Rheu, France (X: 48.099998; Y: −1.8). MRI raw data were collected at UR OPAALE, INRAE, Rennes, France (X: 48.1282588; Y: −1.7007542). Leaf water relations were measured at UR OPAALE while tuber biochemistry was performed at UMR IGEPP, except starch analyzes which was done at UR PSH, INRAE Avignon (X:43.91539, Y:4.87846). Gene expression data were collected at CMI, Saint Malo, France (X: 48.643017; Y: −2.009258).
Data accessibility	Repository name: Data INRAE Data identification number: doi.org/10.57745/FPGDRU Direct URL to data: … https://entrepot.recherche.data.gouv.fr/dataset.xhtml?persistentId=doi:10.57745/FPGDRU
Related research article	Musse M, Hajjar G, Radovcic A, Ali N, Challois S, Quellec S, Leconte P, Carillo A, Langrume C, Bousset-Vaslin L, Billiot B, Jamois F, Joly G, Deleu C and Leport L. Growth kinetics, spatialization and quality of potato tubers monitored in situ by MRI - long-term effects of water stress. Physiologia Plantarum, 2024, 176, e14322. https://doi.org/10.1111/ppl.14322 .

## 1 Value of the Data

• These data describe the complex long-term effects of realistic variable water stress on the potato plant, both above and below ground. They provide new insights and understanding of the effects of water stress on tuber growth and quality.
• These data are a useful resource for biologists, agronomists and breeders interested in the potato crop.
• Researchers interested in developing new image processing methods can use MRI data to create segmentation algorithms.
• All data can be used to improve databases on tuber development and quality and to feed and validate mathematical models.

## 2 Background

Potato is a widespread food crop grown in a wide range of climatic conditions. It is a drought-sensitive crop, and the consequences of drought include a reduction in the number of tubers, a decrease in tuber size, and the appearance of defaults, all of which lead to yield loss. The response of the potato plant to water deficit is complex, and varies according to genotypic factors and the severity, duration, and phenological timing of the stress. Understanding the adaptive responses in potato is a major challenge in agronomy and plant breeding. To achieve this, it is necessary to gather data that describe the functioning of the plant at both the above and below ground levels, including non-invasive spatial and temporal monitoring of tuber formation and growth in the soil. A dataset including MRI, water relations, biochemistry and gene expression data to analyze the effects of water stress on potato plant reported in this datapaper contributes to this advancement. It is related to the research paper “Growth kinetics, spatialization and quality of potato tubers monitored in situ by MRI - long-term effects of water stress” [1] and allows for advanced analyzes by biologists, agronomists and breeders with an interest in the potato crop.

## 3 Data Description

This dataset [2] includes the original and processed MRI images describing the growth kinetics and spatialization of individual tubers belonging to potato plants (Rosanna cultivar of *Solanum tuberosum*) cultivated in pots and subjected to two water regimes and physiological, biochemical and gene expression data measured on leaves and tubers. All these data contribute to highlight long-term effects of water stress on the growth kinetics, spatialization and quality of potato tubers. The measurement time is expressed in days after shoot emergence (DASE). A well-watered regime (control) and a variable water deficit (VWD) regime were indicated by the letters T and S respectively. Four biological replicates (U, V, W and X) were analyzed from plants grown in 4 independent plots. For pH/Eh measurements on tubers, 1 or 3 plants from 3 plots were used to provide 3 or 9 biological replicates. The full plant phenotyping described in this study started at 10 DASE and continued until harvest, which corresponded to 75 DASE. Note that tubers were not detectable before 26 DASE.

The dataset consists of 3 compressed files and 7 tables (Excel files). The 3 compressed files correspond to (i) the original magnetic resonance (MRI) images, (ii) the Avizo software (Avizo3D Pro 2022.1) projects, and (iii) two tables resulting from the images processing using the Avizo software. The 7 tables correspond to data on 1) leaf water potential, 2) leaf water deficit, 3) leaf gene expression, 4) tuber fresh weight, 5) tuber pH and Eh and 6) tuber carbohydrate content, 7) MRI tuber volume and fresh weight obtained at harvest.

### 3.1 MRI images

The first compressed file (“7-MRI-Images.zip”) contains original MRI images in DICOM format consisting of a header with acquisition parameters and image data sets packed into a single *.dcm file. There are 56 folders corresponding to: two water regimes (T and S) X 7 measurement times (J1 to J7 corresponding to 19, 30, 44, 53, 59, 66 and 74 DASE) X 4 repetitions (U, V, W and X). The nomenclature used to describe the folders was water.regime-repetition-measurement.time (example: S-U-J1). Each folder contains about 240 images. The original MRI images are provided at: https://doi.org/10.57745/A639HB .

### 3.2 Tuber volume and position

The compressed files “8-MRI-Processing-tuber-volume-position.zip” correspond to an Avizo project, which consists of a combination of a project file (extension *.hx) and the associated files (folder with the same name than the project file with the extension “-files”). They are provided at: https://doi.org/10.57745/FKQGGY. Associated files are the data that the Avizo software needs to process images and compute the results. In this folder, the names of the Avizo projects follow the same nomenclature as 3D MRI images (example: the project file “S-U-J1.hx” and the folder “S-U-J1-files” allow the segmentation and the volume and position measurement of the tubers in the images sequence contained in the image folder “S-U-J1”). Finally, 1 table (8-MRI-Result-tuber-volume-position.xlsx), obtained from the image processing, corresponds to the data describing the volume and position of the tubers segmented with the Avizo project. The results of the analyzes, provided at https://doi.org/10.57745/MQBMZ9 are organized in 9 columns:

- The code corresponding to the name of the sample is set on the first column (following the nomenclature described for MRI images, example: S-U, or T-W),
- DASE corresponds to the measurement times: 19, 30, 44, 53, 59, 66 and 74,
- Materials is the label used in Avizo projects,
- Tuber is the digital code of each tuber of a plant (example T05), with TM corresponding to the mother tuber,
- Volume3d is the computed volume of the 3D segmented object in mm^3^,
- Area3d is the computed surface area of the 3D segmented object in mm^2^,
- BaryCenterX, BaryCenterY and BaryCenterZ are the positions, in mm, of the barycenter of the 3D segmented object.

### 3.3 Volume and position of internal tuber defaults

The compressed files “9-MRI-Processing-internal-defaults-volume-position.zip” correspond to an Avizo project, consisting of a combination of a project file (extension *.hx) and the associated files (folder with the same name than as the project file with the extension “-files”). They are provided at: https://doi.org/10.57745/PEDBPJ. Associated files are the data that the Avizo software need to process images and compute the results. In this folder, the names of the Avizo projects follow the same nomenclature as the 3D MRI images, with “-defaults” extension (example: the project file “S-U-J1-defaults.hx” and the folder “S-U-J1-defaults-files” allow the segmentation and the volume and position measurement of the internal defaults of the tubers in the images sequence contained in the image folder “S-U-J1”). Only the projects where internal defaults were observed and segmented are presented. Finally, 1 table (9-MRI-Result-internal-defaults-volume-position.xlsx), obtained from the image processing, corresponds to the data of the internal defaults of the tubers segmented with the Avizo project in the dataset. The results of the analyzes, provided at https://doi.org/10.57745/ZQOP2S, are organized in 9 columns:

- The code corresponding to the name of the sample is set on first column (following the nomenclature described for MRI images, example: S-U, or T-W),
- DASE corresponds to the measurement times: 19, 30, 44, 53, 59, 66 and 74,
- Materials is the label used in Avizo projects,
- Tuber is the digital code of each tuber of a plant (example pdt05),
- Volume3d is the computed volume of the 3D segmented object in mm^3^,
- Area3d is the computed surface area of the 3D segmented object in mm^2^,
- BaryCenterX, BaryCenterY and BaryCenterZ are the positions, in mm, of the barycenter of the 3D segmented object.

For internal defaults, since there may be several defaults or group of defaults in tubers, the data per tuber may be present on several rows (example for materials pdt01_01 and pdt01_02).

### 3.4 MRI tuber volume and fresh weight

The “MRI tuber volume & fresh weight” dataset contains 2 columns of data corresponding to the volume (in cm^3^) and fresh weight (in g) of tubers analyzed by MRI of T-plants and 50 rows of data corresponding to 4 replicates (U, V, W and X), with individual tuber measurements (11 for T-U plant, 14 for T-V plant, 11 for T-W plant and 14 tubers for T-X plant). The dataset relating to MRI tuber volume and fresh weight is provided at: https://doi.org/10.57745/KNFUWE.

### 3.5 Leaf water potential

The “Leaf water potential” dataset contains 44 data expressed in MPa, corresponding to 2 water regimes (T and S) x 4 replicates (U, V, W and X), with 4 measurement times for plants grown under T conditions (10, 17, 32 and 46 DASE) and 6 measurement times for plants grown under S conditions (17, 24, 26, 37, 40, 51 and 54 DASE). Leaf water potential under S conditions was measured at the end of the 3 water withholding periods (24, 37 and 51 DASE) and at the end of the 3 watering periods (26, 40 and 54 DASE). Note that after 54 DASE, measurements were stopped due to top-kill at 58 DASE. The dataset relating to leaf water potential is provided at: https://doi.org/10.57745/RAZJHW.

### 3.6 Leaf water deficit

The “Leaf water deficit” dataset contains 5 columns of data expressed in %, corresponding to 5 leaf ranks (LR-1, LR0, LR1, LR2 and LR3) and 40 rows of data corresponding to 2 water regimes (T and S) x 4 replicates (U, V, W and X), with 4 measurement times for plants grown under T conditions (10, 17, 32 and 46 DASE) and 6 measurement times for plants grown under S conditions (24, 26, 37, 40, 51 and 54 DASE). Leaf water deficit under S conditions was measured at the end of the 3 water withholding periods (24, 37 and 51 DASE) and at the end of the 3 watering periods (26, 40 and 54 DASE). Note that after 54 DASE, measurements were stopped due to top-kill at 58 DASE. The dataset relating to leaf water deficit is provided at: https://doi.org/10.57745/8FDS63.

### 3.7 Leaf gene expression

The “Leaf gene expression” dataset contains gene expression data of 7 selected drought-responsive genes expressed in leaves of stressed (S) plants, given as the ratio between S and T plant leaf sample values. This dataset contains 7 columns of data corresponding to the 7 selected genes (DHN1, TAS14, ERD7, RD22, DREB2, AREB2, P5CS) and 24 rows of data corresponding to 4 replicates (U, V, W and X), and 6 measurement times (24, 26, 37, 40, 51 and 54 DASE). The measurement times correspond to the end of the 3 water withholding periods (24, 37 and 51 DASE) and the end of the 3 watering periods (26, 40 and 54 DASE). Note that after 54 DASE, measurements were stopped due to top-kill at 58 DASE. The dataset related to leaf gene expression is provided at: https://doi.org/10.57745/HZKEGW.

### 3.8 Tuber fresh weight

The “Tuber fresh weight” dataset contains data on the total tuber fresh weight (in g). Data were obtained from the tubers harvested from the plant at different measurement dates. Only tubers with a diameter above 15 mm (volume ∼ 2 cm^3^) were included as they were considered for tuber yield. The tubers are numbered according to their final size at 75 DASE, with T01 being the tuber with the highest fresh weight. There are 44 data corresponding to 2 water regimes (T and S) x 4 replicates (U, V, W and X), with 4 measurement times for plants grown under T conditions (32, 46, 68 and 75 DASE) and 7 measurement times for plants grown under S conditions (26, 37, 40, 51, 54, 68 and 75 DASE). Tuber fresh weight data under S conditions were collected at the end of the last 2 water withholding periods (37 and 51 DASE) and at the end of the different watering periods (26, 40 and 54 DASE). Under both T and S conditions, irrigation was stopped from top-kill (58 DASE) to final tuber harvest (75 DASE). Note that no tubers were detectable before 26 DASE. The dataset relating to tuber fresh weight is available at: https://doi.org/10.57745/I7WH97.

### 3.9 Tuber pH and Eh

The “pH of Tubers” dataset contains 2 columns of data corresponding to pH and redox potential (Eh, in mV) values and 42 rows corresponding to 4 measurement times (37, 40, 51, 54 DASE) and 2 water regimes (T and S) x 3 (1 to 3 for DASE 37 and 51) or 9 replicates (1 to 9 for DASE 40 and 54). Under both T and S conditions, irrigation was stopped from top-kill (58 DASE) to final tuber harvest (75 DASE). Note that no tubers were detectable before 26 DASE. The dataset related to tuber pH and Eh is provided at: https://doi.org/10.57745/Z1TNQB.

### 3.10 Tuber carbohydrate content

The “Tuber carbohydrate content” dataset contains 5 columns of data, expressed in % DW, corresponding to starch, glucose, fructose, sucrose and myo-inositol content and 44 rows of data corresponding to 2 watering regimes (T and S) x 4 replicates (U, V, W and X), with 4 measurement times for plants grown under T conditions (32, 46, 68 and 75 DASE) and 7 measurement times for plants grown under S conditions (26, 37, 40, 51, 54, 68 and 75 DASE). Tuber carbohydrate data under S conditions were collected at the end of the last two water withholding periods (37 and 51 DASE) and at the end of the different watering periods (26, 40 and 54 DASE). Under both T and S conditions, irrigation was stopped from top-kill (58 DASE) to final tuber harvest (75 DASE). Note that no tubers were detectable before 26 DASE. The dataset related to carbohydrate content in tubers is provided at: https://doi.org/10.57745/AQWM87.

## 4 Experimental Design, Materials and Methods

### 4.1 Plant material

The study was performed on potato plants (Rosanna cultivar of *Solanum tuberosum*) provided by Germicopa (Quimper, France). The day of shoot emergence (DASE 0), defined as the time when 50 % of the plant shoots could be observed, corresponded to May 3, 2021. The plants were grown in 25 L plastic pots (Airpot®, 27 cm diameter, 50 cm high) filled with a sandy soil (Falienor® ref. 992016F1) from which metal particles were removed, in a INRAE greenhouse (Le Rheu, France) equipped with an air-cooling system. The average day/night temperatures and relative humidity were 22.0 °C (+/− 1.2 °C) / 11.4 °C (+/− 0.9 °C) and 59.9 % (+/− 5.0 %) / 90.1 % (+/− 1.5 %).

### 4.2 Watering regimes

During tuber growth and maturation, from 10 to 58 DASE, the experimental design included two different watering regimes: (i) a well-watered regime in which soil moisture was maintained at 70 % of field capacity (T) and (ii) a variable water-deficient regime (S) in which soil moisture was reduced to 20 % of field capacity at 24, 37 and 51 DASE, followed each time by a three- to four-day period of rehydration to 70 % of field capacity. The amount of water to be supplied corresponded to approximately 100 % and 30 % of evaporative demand under the T and S regimes respectively. The quantity of water to be supplied was determined by daily manual weighing. For both watering regimes, irrigation was stopped from top-kill (58 DASE) to final tuber harvest (75 DASE).

### 4.3 Plant sampling

As MRI is a non-invasive and non-destructive technique, the same four plants from each regime (4 T and 4 S) were imaged over the 2-month period from the onset of tuber growth (17 DASE) to final harvest (75 DASE). Fifty-six plants (24 T and 32 S) were used for destructive physiological measurement on leaves (water potential and deficit), leaf gene expression and tuber carbohydrate quantification. Dedicated organs (leaves and/or tubers) were sampled at the different DASE. In addition to these plants, 42 plants (18 T and 24 S) were dedicated for redox potential (Eh) and pH measurements.

### 4.4 Leaves

At 10 DASE, for all plants, the youngest leaf (larger than 2 cm emerging from the apical stem was tagged with a plastic wire and was allocated to rank 0 (LR0). The leaves with 1, 2, 3, 4 ranks below and 1, 2 and 3 ranks above LR0 were assigned to rank −1 (LR-1), −2 (LR-2), −3 (LR-3), −4 (LR-4), +1 (LR1), +2 (LR2) and +3 (LR3), respectively. Leaves sampled from the fourth rank from the youngest leaf (LR-4 at 10 DASE to LR-1 at 54 DASE) were used for leaf water potential measurements while leaf discs collected from two leaflets for five leaf ranks (LR-1 to LR3) were used for leaf water deficit measurements. Leaves sampled from the largest leaflet of the LR0 leaf were harvested and frozen for RNA extraction.

### 4.5 Tubers

From 26 to 75 DASE, all harvested tubers were ranked according to their fresh weight (FW). The first tuber (with the highest FW) was assigned to rank 1 (T01), the second to rank 2 (T02) and so on. For each plant, all tubers larger than 15 mm were weighted to determine the total tuber fresh weight. T02 was used to determine water and carbohydrate content. T0 tuber of dedicated plants was used for pH and Eh measurements.

### 4.6 MRI

MRI measurements were carried out with a 1.5T MRI scanner (Magnetom, Avanto, Siemens, Erlangen, Germany) equipped with a circularly polarized head array coil. In subsequent experiments, the pots were placed horizontally in the MRI tunnel in the same position. Measurements of the underground parts (24 cm depth) of the potato plants were performed at room temperature using a 3D Fast Recovery Turbo Spin Echo (FR-TSE) sequence with the following acquisition parameters: 320 × 320 × 324 imaging matrix and 256 mm × 256 mm x 324 mm field of view, resulting in a resolution of 0.8 mm × 0.8 mm × 1 mm, flip angle 170 °, repetition time 100 ms, effective echo time 28 ms, echo spacing 9.5 ms, echo train length 6, bandwidth 200 Hz/pixel, 43 % interpolation in the slice direction, 1 average. The acquisition time was 18 min per plant.

At 75 DASE, tubers of T plants were harvested and their fresh weight was determined using an electronic precision balance (0.1 mg).

Image processing was carried out using the Avizo 3D Pro software (FEI Company, version 2021.2). Semi- automatic tuber segmentation was performed by first applying a classical threshold set by the operator, followed by a morpho-mathematical operation (shrink+grow) applied to remove some of the effects of noise. Manual intervention was then required to separate any stuck tubers and to separate the tubers from the roots. A unique label was then assigned to each isolated tuber and was used to determine the barycenter and volume of each tuber for the period analyzed. Only tubers with a diameter greater than 3 mm were included in the analysis.

The volume of defaults in each individual tuber for the period analyzed was estimated using an approach similar to that used for tuber segmentation, except that defaults were segmented directly prior to labeling.

For both tuber and defaults analysis, the images were processed in reverse date order of measurement (starting from the end of the growth period) in order to facilitate the segmentation of tubers/defaults in the images acquired at the earlier time points.

### 4.7 Plant physiology, biochemistry and gene expression analyzes

Data on leaf water relations and gene expression and tuber biochemistry were obtained using an electronic precision balance (0.1 mg) for leaf water deficit and tuber fresh weight, a WP4C dewpoint potential meter (Meter Group Inc., Pullman, WA, USA) in “precise mode” for leaf water potential, a LAQUAtwin-pH-22 m (HORIBA Advanced Techno, Kyoto, Japan) for tuber pH and an Ag/AgCl reference electrode radiometer analytical E21M002 and platinum plate(5 × 5 mm M241 Pt) radiometer analytical electrode (Hach, Loveland, CO, USA), with a (10 × 106 Ohm input resistance) Voltcraft VC850 multimeter (Conrad, Hirschau, Germany) for tuber Eh.

Leaf water deficit data were measured according to [3] as 1-(FW-DW)/(TW-DW) where FW is fresh leaf weight, TW is leaf weight after soaking in water for 1 h (T) or 4 h (S), and DW is leaf dry weight after 48 h in a vacuum steam room at 70 °C.

Redox potential was measured in a 1 cm thick central cross section of tubers as described in [4]. Electrodes were inserted into the flesh of the tubers at a distance of 5 mm. The value retained corresponded to that displayed on the multimeter which remained stable for one minute. After measurement according to Ag/AgCl reference electrode, all redox potentials were transformed to give Eh according to the “normal hydrogen electrode”. The redox electrodes were calibrated before the first measurement and every 10–12 measurements, with Mettler Toledo redox buffer solution 220 mV (pH = 7) composed of potassium hexacyanoferrate (III), potassium hexacyanoferrate (II), potassium dihydrogen phosphate and disodium hydrogen phosphate. All measurements were conducted outdoor, in an environment identified as being free of electromagnetic interference. Once the Eh was measured, the tuber slice was cut out and grinded. The mixture was filtered through cotton wool and the juice was squeezed onto a Horiba LAQUAtwin-pH-22 m for pH measurement.

For starch analysis, the soluble compounds were removed from the tuber dry powder using a methanol-water-chloroform solution. The remaining pellet was enzymatically digested [5], and the resulting glucose was measured enzymatically in a microplate reader.

For metabolite analysis, 10–15 mg of freeze-dried powder were sampled for methanol-chloroform-water based extraction adapted from [6]. Samples were suspended in 400 μL of methanol containing 400 μM Xylitol as internal standards for GC quantification. Samples were gently agitated for 15 min at room temperature. Then, 250 μL of chloroform was added followed by 10 min of agitation and a final addition of 500 μL of water. Samples were vortexed vigorously and centrifuged at 12,000 g for 5 min. The supernatant (500–700 μL) was collected and an aliquot of 50 μL was independently vacuum-dried for 2 h before metabolite profiling.

Methods for the analysis of sugars were adapted from [6,7]. Briefly, dry residues were resuspended in 50 μL of 20 mg.mL^−1^ methoxyamine hydrochloride in pyridine at 40 °C for 60 min. Then, 50 μL of MSTFA (N-methyl-trimethysilytrifluoroacetamide) was added for derivatization (30 min at 40 °C). One μL of the mixture was injected into a gas chromatography-flame ionization detector system (6890N GC-FID, Agilent Technologies, Santa Clara, CA, USA) equipped with MultiPurposeSampler MPS Robotic Pro autosampler (Gerstel, Mülheim, Germany), a split/splitless injector (split mode set to 1:20) at 260 °C, a TG 5MS 30 *m* × 0.32 mm × 0.25 mm column and a flame ionization detector at 310 °C. The temperature gradient was as follows: 4 min at 70 °C, 10 °C.min^−1^ until 198 °C followed by 2 min at 198 °C, 1 °C.min^−1^ until 202 °C, 15 °C.min^−1^ until 268 °C followed by 3 min at 268 °C, 1 °C.min^−1^ until 272 °C, 10 °C.min^−1^ until 310 °C and finally 7 min at 310 °C. Chemstation software (Agilent Technologies, Santa Clara, CA, USA) was used for data processing. External standards of known concentration were run every 10 samples to calibrate the system. Compounds were quantified with to the internal standard signal xylitol. Metabolite contents (glucose, fructose, sucrose and myo-inositol) were expressed as % of dry matter (DM).

Leaf gene expression was quantified by qRT-PCR using a Universal SYBR Green Supermix (Bio-Rad, CA, USA) in a real-time PCR detection system (Bio-Rad, CA, USA). The qRT-PCR reactions were obtained in technical triplicates using independent cDNA reactions for each biological replicate and 300 nM of gene-specific primer pairs (Table 1). The thermal cycler protocol included preincubation at 98 °C for 3 min, first followed by 40 cycles of amplification, each consisting of denaturation at 98 °C for 15 s, then by annealing at 60 °C for 30 s and then by elongation at 72 °C for 15 s with a final 5-min extension at 72 °C. Additionally, melting curve analysis was performed at the end of each assay to confirm the absence of multiple products or primer dimers. The expression of all genes analyzed was normalized to two potato reference genes, namely, StRPL2 and StEF1α.

**Table 1:** List of analyzed genes and corresponding primers used for qRT-PCR.

GENE	ACCESSION NO.	FORWARD PRIMER	REVERSE PRIMER	AMPLICON SIZE (BP)
**StAREB2**	XM_015312047	5′-CAGAACCATCAACCACAGCA-3′	5′-ATACCAACCATCCCTACCCTC-3′	143
**StDREB2**	JN125858	5′-AAAGCAGAGGGAACACCAAC-3′	5′-GGGAAGAATAAGAACCAAGCCA-3′	128
**StDHN1**	XM_015304546	5′-AGGAGAAATTGCCAGGAGGT-3′	5′-GTGCCTTCCATACCATAACCAG-3′	85
**StTAS14**	XM_015304540	5′-TGGCACTCAAGGTAGCGG-3′	5′-TCCTCCTCCTGGCATCTTCT-3′	175
**StERD7**	XM_006359626	5′-TGGGGATGTTACTGTGGATAGG-3′	5′-GAGACCTTCACTACACCTGAGA-3′	180
**StRD22**	JX839749	5′-CACACAGTTAGCAAGAGCAAAG-3′	5′-GGTATCCAAGTGACAAACAGCA-3′	93
**StP5CS**	XM_006346765	5′-CTTGTTGAAAGGAGGGAAGGAG-3′	5′-CAGTTGGAGGAATGGATGAGG-3′	80
**StRPL2**	DQ252497	5′-GAGGGAGAGAGAGAAGAGAGAG-3′	5′-GGTGGTGGGTATGGGATTTG-3′	100
**StEF1α**	AB061263	5′-GATGATTCCCACCAAGCCCA-3′	5′-TGACAACACCGACAGCAACA-3′	107

## Limitations

Despite the large number of unique features, the dataset is somewhat limited by its size. The relatively low number of biological replicates resulted in data dispersion for some destructive measurements (i.e. leaf gene expression, pH, Eh, starch, glucose and fructose contents).

## Ethics Statement

The authors have read and follow the ethical requirements for publication in Data in Brief and confirm that the current work does not involve human subjects, animal experiments, or any data collected from social media platforms.

## CRediT Author Statement

Maja Musse and Laurent Leport designed the study; Ghina Hajjar and Stéphane Quellec carried out the MRI experiments; Ghina Hajjar and Sylvain Challois performed the MRI image analyzes; Carole Deleu and Laurent Leport set up the plant experimental design; Aël Radovcic, Patrick Leconte, Aurélien Carillo and Christophe Langrume performed data collection and/or physiological analyzes; Nusrat Ali, Bastien Billiot and Frank Jamois performed gene expression analyzes; Lydia Bousset-Vaslin performed pH/Eh analyzes; Solenne Berardocco performed metabolite analyzes and Doriane Dumont and Patricia Laugier performed starch analyzes. Maja Musse, Sylvain Challois and Laurent Leport organized and wrote the datapaper with contributions from Ghina Hajjar, Nusrat Ali, Lydia Bousset-Vaslin, Sollene Berardocco, Doriane Dumont and all authors approved the final text. Sylvain Challois managed data curation, with contributions from Maja Musse and Laurent Leport.

## Data Availability

MRI, leaf water relation and gene expression and tuber biochemical data for analysis of the effects of water stress on tuber development and quality (Original data) (Data INRAE).
